# Clinical and Neuroradiological Manifestations of Cerebral Amyloid Angiopathy: A Closer Look into the Natural History of a Frequent Disease

**DOI:** 10.3390/jcm14051697

**Published:** 2025-03-03

**Authors:** Marialuisa Zedde, Fabrizio Piazza, Rosario Pascarella

**Affiliations:** 1 Neurology Unit, Stroke Unit, Azienda Unità Sanitaria Locale-IRCCS di Reggio Emilia, Viale Risorgimento 80, 42123 Reggio Emilia, Italy; 2 CAA and AD Translational Research and Biomarkers Laboratory, School of Medicine and Surgery, University of Milano-Bicocca, Via Cadore 48, 20900 Monza, Italy; fabrizio.piazza@unimib.it; 3 Neuroradiology Unit, Azienda Unità Sanitaria Locale-IRCCS di Reggio Emilia, Viale Risorgimento 80, 42123 Reggio Emilia, Italy; pascarella.rosario@ausl.re.it

**Keywords:** cerebral amyloid angiopathy, CAA, small vessel disease, SVD, cortical superficial siderosis, WMHs, convexal non-aneurysmal subarachnoid hemorrhage, CAA-related inflammation, ARIA, AD

## Abstract

Cerebral amyloid angiopathy (CAA) is one of the most prevalent small vessel diseases (SVDs). Its neuroradiological hallmarks are both hemorrhagic and non-hemorrhagic ones. Among the clinical manifestations, transient focal neurological episodes (TFNEs) are associated with an increased risk of bleeding in a short time period and with convexal subarachnoid hemorrhage (SAH). The natural history of CAA is incompletely characterized in the literature, because the focus has been mostly on hemorrhagic events, while both clinical and non-hemorrhagic presentations are possible and sometimes underestimated. Furthermore, new diagnostic criteria have incorporated non-hemorrhagic Magnetic Resonance Imaging (MRI) markers and non-hemorrhagic clinical presentations. Disease trajectories are often individual and help provide food for thought and discussion on some issues, thus allowing for a greater and deeper evaluation. We, therefore, present a case that exemplifies how the natural history of CAA can be atypical compared to its expected course, which is long and not only hemorrhagic. Several episodes of CAA-related inflammation, with prevalent, but not exclusive, leptomeningeal involvement, were evaluated and treated in the presented case, in which the intraparenchymal cerebral hemorrhagic manifestation was the last in the patient’s history. CAA may have a very long natural history. During the disease’s course, inflammatory features might be prominent in neuroimaging but not strongly symptomatic, and intraparenchymal cerebral hemorrhage (ICH) may be a late event. The awareness of this subtype of the disease allows us to better explore the pathophysiology of CAA and to increase the level of clinical suspicion for the diagnosis. Furthermore, the distinction between different disease phenotypes can provide useful information for patient management in clinical practice.

## 1. Introduction

Small vessel diseases (SVDs) are a frequent cause of ischemic stroke, intracranial hemorrhage, and vascular cognitive impairment [1,2,3]. They are a highly prevalent category of cerebrovascular disorders with an age-dependent distribution and a frequent incidental finding in neuroimaging studies in population cohorts [4]. The main characterization of SVDs is on clinical, neuropsychological, biochemical, and neuroradiological issues [4,5,6]. This last issue is composed of a well-defined set of Magnetic Resonance Imaging (MRI) signs, present with variable combinations in all SVD patients across the lifespan and the natural history of the disease [5,6]. In addition, MRI signs of SVD support the differentiation between two main categories, i.e., deep perforator arteriopathy (also commonly called hypertensive arteriopathy) and cerebral amyloid angiopathy (CAA) [4,5,6,7]. The main features of both SVDs are partially overlapping, and one of the differences in MRI patterns is the deep and strictly lobar location of cerebral microbleeds (CMBs) in the first and second one, respectively [7]. In addition, mixed SVDs are possible [8], and one of the main drivers of bleeding risk in CAA, i.e., cortical superficial siderosis (cSS), is absent in deep perforator arteriopathy [5,6,9,10]. CAA has received a lot of interest in recent decades because of its burden and morbidity and because of its association with Alzheimer’s disease (AD) and the potential therapeutical implications [10]. In fact, one of the main adverse events in immunotherapy trials for AD is the occurrence of an inflammatory process in the brain with a typical neuroradiological pattern, called ARIAs (amyloid-treated imaging abnormalities). ARIAs include both inflammatory (ARIA-E for exudate) and hemorrhagic markers (ARIA-H) characterized by the new appearance in patients treated by immunotherapy and in a small proportion of patients taking placebo [11,12,13,14,15,16,17,18]. Then, an ARIA is not only an iatrogenic phenomenon, and the similarities with a spontaneous counterpart described in CAA patients (CAA-related inflammation) are evident in MRI appearance and presumed biological processes [19,20,21,22,23]. In the natural history of the vascular disease (CAA), some issues did not receive attention, the follow-up being relatively short and focused on individual events (bleeding, mainly intracerebral hemorrhage or ICH and recurrent CAA-related inflammation in some series). In particular, the recurrence of convexity (or sulcal) subarachnoid hemorrhage (SAH) and the relationship among SAH, CAA-related inflammation, and transient focal neurological episodes (TFNEs) have not been clearly described [9]. At the same time, the global increase in the SVD burden of individual patients received little attention outside some population studies focusing on white matter hyperintensities (WMHs) and lacunar lesions [5,6]. Separate diagnostic criteria are available for CAA, including cortical SAH as a bleeding event, CAA-related inflammation, and ARIAs, but little is available to define the long-term history of the disease [24,25,26]. The main aim of this review is to present the long-term natural history and evolution of CAA in an individual patient across more than a decade, taking into account the relative weight of the inflammatory component with respect to the chronic increase in the SVD burden and acute bleeding occurrence. In addition, a neuroradiological phenotype with isolated or prominent leptomeningeal involvement has not been strongly established in CAA-related inflammation. The recurrence of inflammatory events has been considered a rare occurrence, but their incidence is probably underestimated. In fact, some of the published case series share the limitation of a retrospective design and unstructured follow-up.

## 2. December 2013: Sulcal SAH and TFNEs

**Figure 1:**
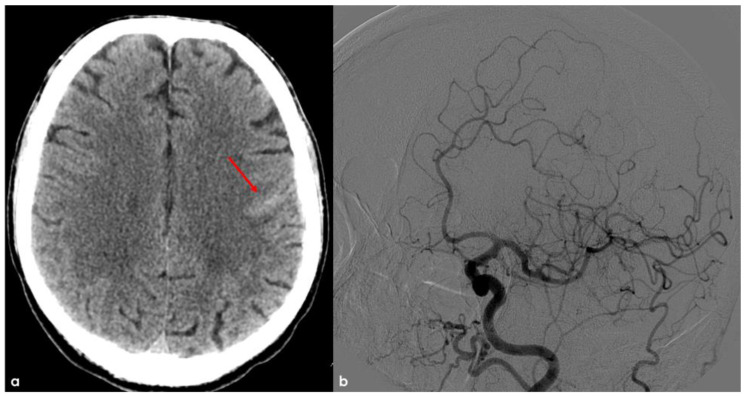
Brain NCCT (panel (**a**)) showing a linear hyperdensity within the left precentral sulcus, suggestive of convexal SAH (red arrow). In panel (**b**), digital subtraction angiography (DSA) from left common carotid artery injection in oblique view is provided in the arterial phase, excluding the arterio-venous shunt.

In December 2013, the patient was a 68-year-old man, with an unremarkable past history, and he came to the Emergency Department (ED) because of the sudden occurrence of very short episodes of right “positive” hemisensory syndrome (paresthesias) and a progressing course through the right lips, arm, and leg. These episodes lasted 1 to 3 min and occurred from 2 to 5 times a day. The brain non-contrast Computed Tomography (NCCT) showed a linear hyperdensity in the precentral sulcus as for cortical SAH, without a recent or past history of trauma (Figure 1). The main differential diagnoses of spontaneous cortical SAH were excluded, and CT angiography (CTA) and subsequent digital subtraction angiography (DSA) showed normal findings.

Spontaneous non-aneurysmal convexity subarachnoid hemorrhage (cSAH) is a distinct type of subarachnoid bleeding localized to the cortical surface. Unlike other forms of SAH, it remains confined and does not spread into the sylvian or hemispheric fissures, basal cisterns, brain parenchyma, or ventricular system. The etiology of cSAH differs based on patient age [27]. In younger individuals, the primary causes include reversible cerebral vasoconstriction syndrome (RCVS), which may or may not be associated with posterior reversible encephalopathy syndrome (PRES), as well as cerebral venous thrombosis (CVT) and other uncommon conditions [28,29]. In contrast, cerebral amyloid angiopathy (CAA) is the most prevalent cause in patients over 60 years old [28,30]. Importantly, CAA is now officially acknowledged as a cause of cSAH, independent of its well-established link to intracerebral hemorrhage (ICH), as per the revised Boston 2.0 diagnostic criteria [31]. While CAA-related cSAH often resolves without lasting neurological deficits, it is associated with considerable long-term risks, including a 13% annual incidence of lobar hemorrhage (95% CI 10–17%), an 11% yearly recurrence rate of cSAH (95% CI 8–15%), and a 5% yearly risk of ischemic stroke (95% CI 3–8%) [32]. Additionally, cSAH may present alongside ICH and other CAA-related conditions, such as CAA-related inflammation, which can influence treatment strategies [23,33].

**Figure 2:**
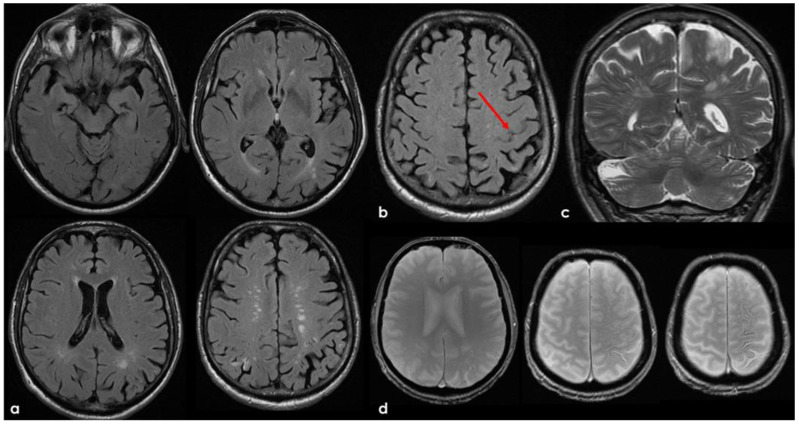
Brain MRI acquired a few days after the NCCT provided in Figure 1. Panel (**a**) shows the Fluid Attenuated Inversion Recovery (FLAIR) axial slices with scattered subcortical white matter hyperintensities in the centrum semiovale on both hemispheres and a covert cortical brain infarction in the right parietal lobe (second row on the right). In panel (**b**), the FLAIR hypointense filling of the left precentral sulcus corresponding with the cSAH in Figure 1 is illustrated (red arrow). Panel (**c**) shows the enlarged perivascular spaces (EPVSs) in the posterior part of the cerebral hemispheres on coronal T2W-MRI. Finally, panel (**d**) shows, on T2* gradient echo (GRE) MRI, the hypointense sulcal signals of a diffuse (more than 3 sulci) superficial siderosis in the left hemisphere.

The clinical presentation was highly supportive for TFNEs, and a brain MRI showed two isolated cortical CMBs and a pattern of diffuse (≥3 sulci) multifocal cSS with enlarged perivascular spaces in the centrum semiovale and mild scattered WMHs in the subcortical white matter (Figure 2).

A key clinical feature of cSAH is transient focal neurological episodes (TFNEs), also known as amyloid spells [31]. These episodes manifest as recurrent, stereotyped neurological symptoms that gradually spread and typically last for several minutes. Recognizing TFNEs is crucial, as they serve as an important diagnostic marker for CAA beyond intracerebral hemorrhage (ICH) and may precede symptomatic ICH. Proper identification of TFNEs helps prevent misdiagnosis as transient ischemic attacks (TIAs), thereby avoiding inappropriate antithrombotic treatment, which could increase the risk of ICH. Although the precise mechanisms underlying amyloid spells remain uncertain, potential causes include vasoconstriction of amyloid-laden small vessels, seizure-like activity from microbleeds, or focal convexity subarachnoid hemorrhages. Among these theories, cortical spreading depression is considered the leading pathophysiological mechanism [31,34]. Evidence suggests a strong association between TFNEs and cortical superficial siderosis (cSS) or cSAH. Studies indicate that CAA patients with TFNEs exhibit a higher prevalence of cSS or cSAH compared to those without TFNEs (50% vs. 19%; *p* = 0.001). Moreover, half of the patients experiencing TFNEs developed symptomatic lobar ICH within a median follow-up period of 14 months. A meta-analysis further highlighted a 24.5% risk (95% CI 15.8–36.9%) of symptomatic ICH occurring within eight weeks of a TFNE, independent of prior ICH or other clinical factors [31]. Longitudinal data reinforce the significance of cSS and cSAH in predicting future cerebral hemorrhagic events. In a cohort of 90 suspected CAA patients with evidence of cSS and/or cSAH, follow-up data were available for 76 patients (84%). Among them, 29% (10 patients) experienced symptomatic cerebral bleeding events, including ICH or cSAH, with a median event time of 21 months and an average of 34 months. In contrast, only one symptomatic event (2.4%) occurred among patients without cSS or cSAH, occurring 25 months after the initial scan (*p* = 0.001) [35].

Further studies have clarified the prognosis of CAA-related cSAH [32]. The annual risk estimates per patient include the following: ICH at 13.2% (95% CI 9.9–17.4%), recurrent cSAH at 11.1% (95% CI 7.9–15.2%), combined ICH or cSAH at 21.4% (95% CI 16.7–26.9%), ischemic stroke at 5.1% (95% CI 3.1–8%), and overall mortality at 8.3% (95% CI 5.6–11.8%). A multivariable analysis revealed that patients with probable CAA faced a significantly higher risk of ICH (HR 8.45, 95% CI 1.13–75.5, *p* = 0.02) and cSAH (HR 3.66, 95% CI 0.84–15.9, *p* = 0.08) compared to those classified as having possible CAA. However, the differences in ischemic stroke risk (HR 0.56, 95% CI 0.17–1.82, *p* = 0.33) and mortality (HR 0.54, 95% CI 0.16–1.78, *p* = 0.31) were not statistically significant.

## 3. May 2014: CAA-Related Inflammation

**Figure 3:**
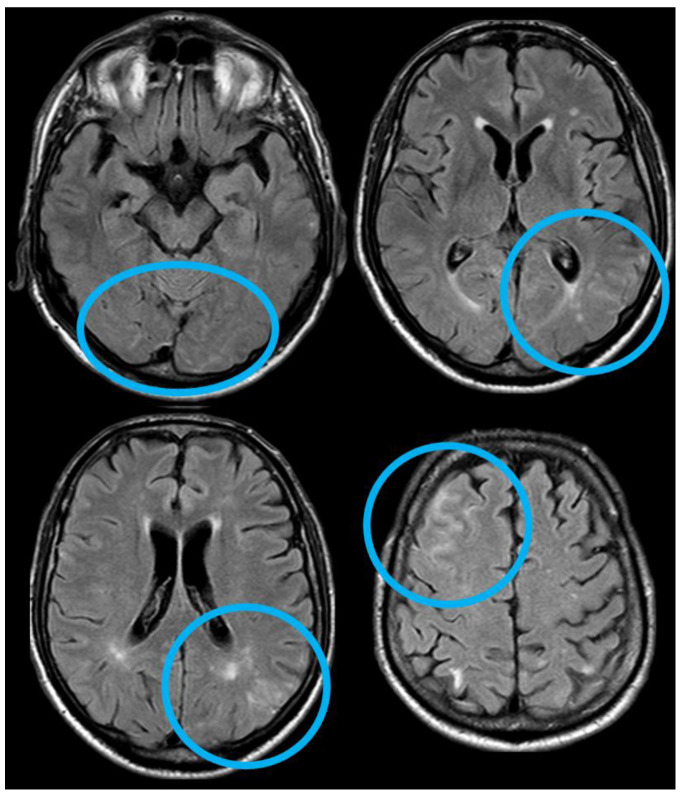
Brain MRI (axial FLAIR) showing spontaneous sulcal hyperintensity involving several sites, in particular, the occipital pole on both sides (left > right), the left temporal-occipital transition, the left parietal lobe, and the right anterior frontal lobe (blue circles).

**Figure 4:**
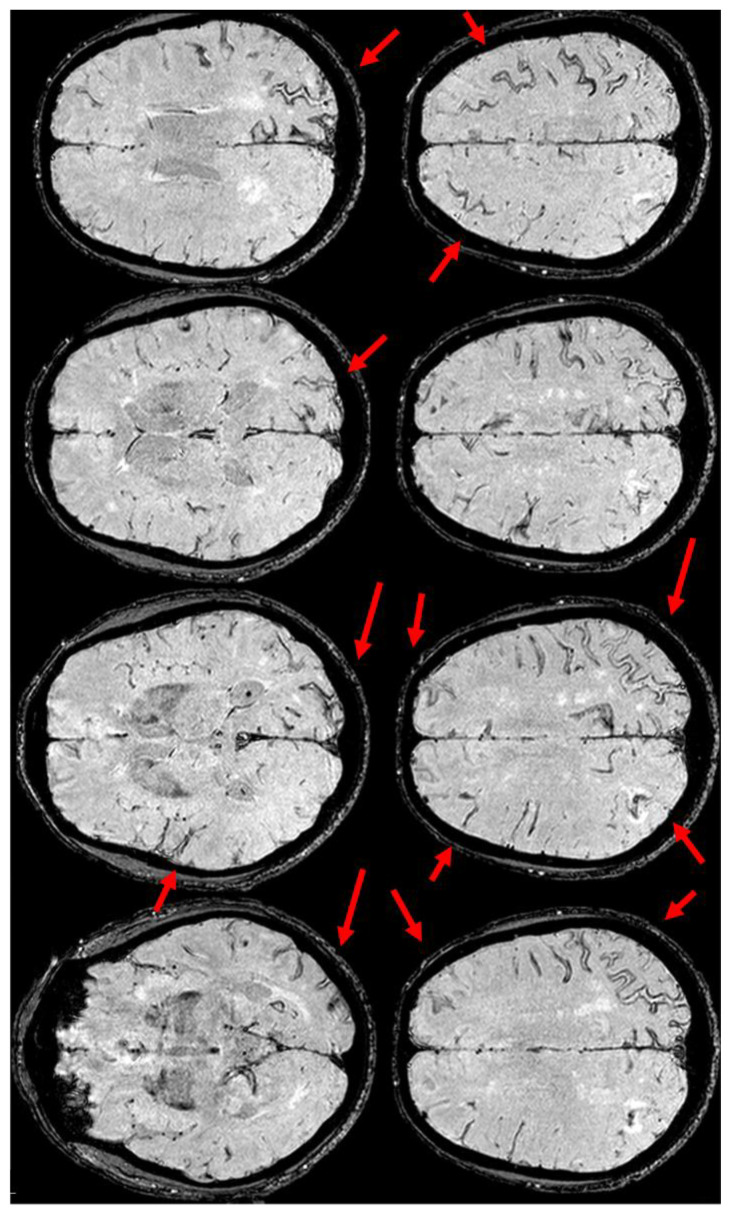
Brain MRI (Susceptibility Weighted Imaging—SWI) in the axial plane, showing the extension of cSS to the left posterior temporal and occipital lobe, to the left posterior parietal lobe, to the parasagittal sulci at the vertex on both sides, and to the right frontal and parietal lobes (red arrows).

After 5 months (May 2014), the patient performed a follow-up brain MRI. In the meantime, no focal neurological symptoms developed, and his unique complaint was a subjective feeling of being unstable in walking. The MRI (Figure 3 and Figure 4) showed spontaneous multifocal sulcal hyperintense signals on FLAIR sequences in the right frontal, left posterior temporal, left posterior parietal, and bilateral occipital lobes with post-contrast enhancement in the same sites. Both T2* and SWI sequences showed a progression of the cSS involving several sulci in the left and right hemispheres.

The neuroimaging pattern of the diffuse sulcal FLAIR hyperintensity in Figure 3, together with the clinical context of the patient and the known and increased microhemorrhagic burden, mainly expressed by cSS with only two isolated cortical CMBs (not shown in the figures), not only supported the diagnosis of cerebral amyloid angiopathy, retrospectively applying the Boston 2.0 criteria [24], but suggested the occurrence of sulcal or leptomeningeal effusion. This phenomenon, called CAA-related inflammation from the CAA side [23], and ARIAs from the AD side (in particular, in immunotherapy trials) [13,15,36,37], is an immune-mediated encephalopathy, whose pathogenesis is still a matter of discussion. In CAA-related inflammation, the role of anti-amyloid autoantibodies has been described, and they could be involved in ARIAs too [21,38]. The frequent coexistence of CAA and AD in the same individual from the pathological reports (nearly 80%), the presence of similar neuroradiological findings in CAA-related inflammation and ARIAs, and the documentation of ARIAs in the placebo arms of immunotherapy trials support the hypothesis that CAA-related inflammation and ARIAs may be different subtypes of the same phenomenon [38]. An ARIA is a dual-component manifestation, i.e., exudation (ARIA-E) and hemorrhage (ARIA-H). The diagnostic criteria of ARIAs rely only on neuroradiological appearance in comparison with previous MRIs of the patients, because of their proposal within clinical trials of immunotherapy in AD. Moreover, an ARIA explicitly includes a pure leptomeningeal exudation, and the severity scores highlight the progression of hemorrhagic load due to the inflammatory process [15,39].

A diagnosis of CAA-related inflammation was made, and in a retrospective revision after the publication of the new diagnostic criteria [26], probable CAA-related inflammation was confirmed.

**Figure 5:**
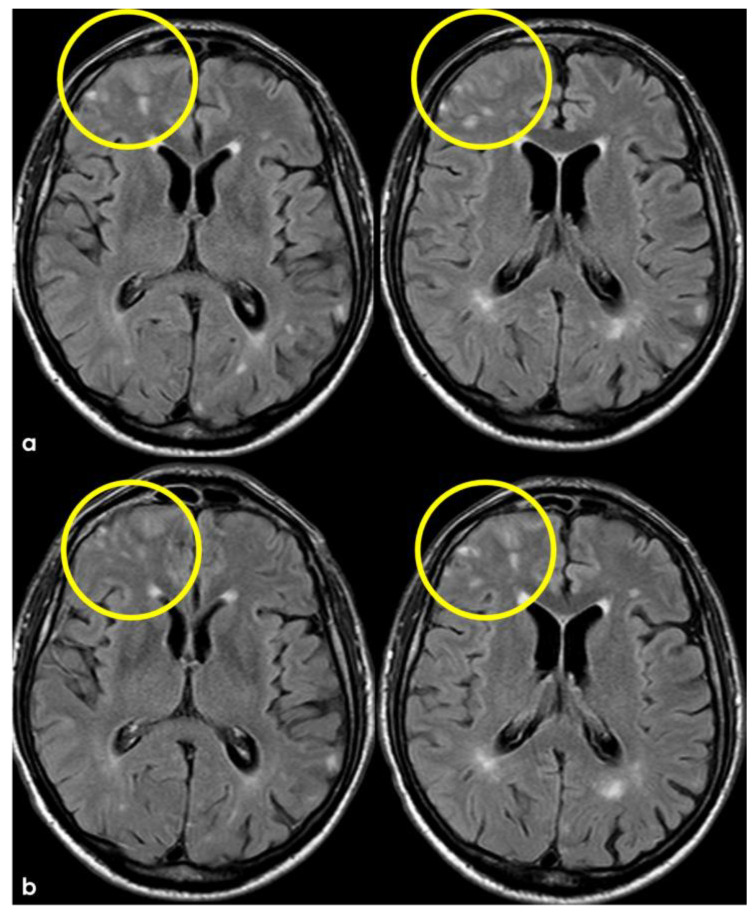
Brain MRI at 3 months from the previous one (Figure 3 and Figure 4), showing in axial FLAIR the persistence of spontaneous sulcal hyperintensity in the left anterior frontal lobe (panel (**a**), yellow circles) with contrast enhancement (panel (**b**), yellow circles). The remaining previously involved lobes (Figure 3) showed resolved sulcal involvement.

The patient was treated using steroids, starting with Methylprednisolone, 1 gr intravenously for 5 days, and continuing with tapering off oral prednisone for several weeks [23]. The follow-up MRI 3 months after steroid treatment showed a strong improvement in the exudative pattern (Figure 5).

**Figure 6:**
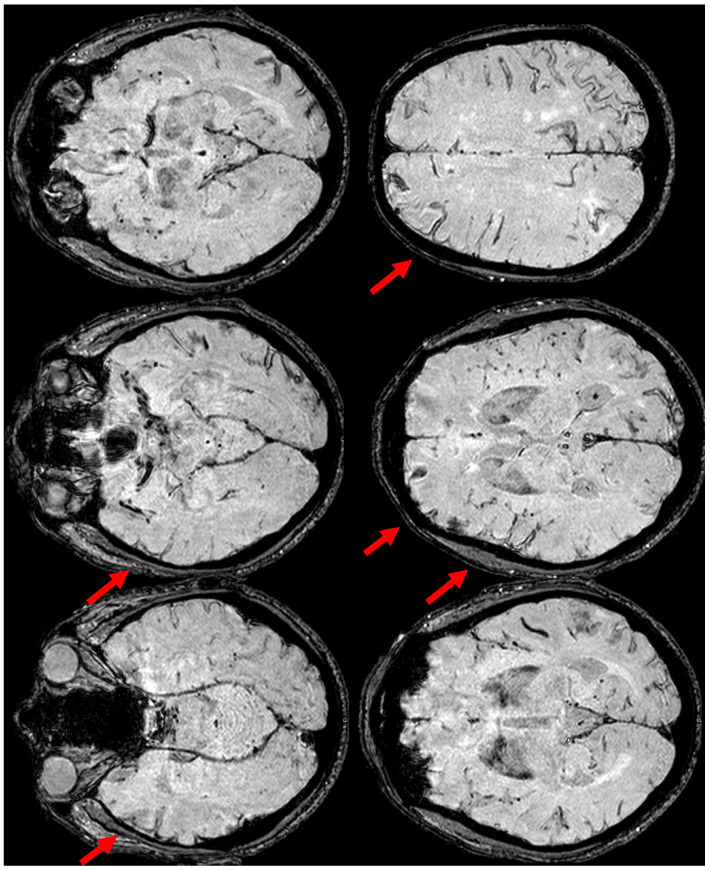
Brain MRI at three months from the start of CAA-related inflammation (Figure 3), showing on axial SWI, the increased extension of cSS, actually involving the left anterior temporal lobe and the left anterior frontal lobe (red arrows). In addition, the involvement of the left frontal sulci is more extensive than in the previous MRI.

At the same time, SWI showed a further progression of the already known cSS, as is often found in an ARIA [13,40] (Figure 6).

**Figure 7:**
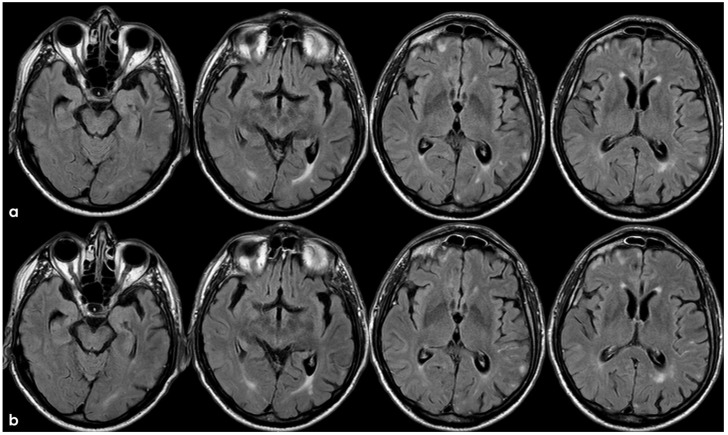
Brain MRI 5 months after the first finding of CAA-related inflammation (Figure 3), showing the resolution of the exudative phase. Panel (**a**) shows the axial FLAIR sequence, and panel (**b**) shows the corresponding post-contrast axial FLAIR slices. The previously found spontaneous sulcal hyperintensities resolved and no contrast enhancement is illustrated.

A follow-up MRI at 5 months from the onset of CAA-related inflammation showed the resolution of the exudative process, leaving cortical-subcortical FLAIR hyperintensities in the left anterior frontal lobe (Figure 7).

The natural history of CAA-related inflammation has been described in a large prospective multicenter cohort within an international network (the inflammatory cerebral amyloid angiopathy and Alzheimer’s disease βiomarkers International Network) [23,41]. A comprehensive protocol for systematic data collection was implemented during initial presentations and subsequent in-person follow-up visits. This study utilized a standardized MRI protocol, including T1-weighted, GRE-T2*, FLAIR, and contrast-enhanced T1 imaging on a 1.5T system at 3, 6, 12, and 24 months. Blinded investigators analyzed all scans to prevent bias. Primary outcomes assessed included survival, clinical and radiologic recovery, incidence of ICH, and recurrence of CAA-related inflammation. The cohort included 113 participants, categorized as 10.6% definite, 71.7% probable, and 17.7% possible CAA-related inflammation, with a mean age of 72.9 years and 43.4% female. APOEε4 gene carriers comprised 37.1%, while 36.3% had Alzheimer’s disease, and 33.6% had prior ICH. Baseline cortical superficial siderosis (cSS) correlated with prior ICH (52.6% vs. 14.3%, *p* < 0.0001) and an increased risk of new ICH (19.3% vs. 3.6%, *p* = 0.009). Clinical recovery was observed in 70.3% (95% CI 61.6–78.5%) within 3 months and 84.1% (95% CI 76.2–90.6%) within 12 months. Radiologic recovery rates were 45.1% (95% CI 36.4–54.8%) at 3 months and 77.4% (95% CI 67.7–85.9%) at 12 months. However, 38.3% (95% CI 22.9–59.2%) experienced at least one recurrence within 24 months. Recurrence was significantly higher in patients with abrupt IV corticosteroid withdrawal compared to gradual oral tapering (HR 4.68, 95% CI 1.57–13.93; *p* = 0.006). Findings from this largest longitudinal CAA-related inflammation cohort underscore the transient but relapsing nature of the condition and highlight the benefits of a gradual corticosteroid tapering strategy. The study also stresses the need for precise differential diagnosis in spontaneous ARIA-like events, particularly in Alzheimer’s patients undergoing immunotherapy.

## 4. December 2015: Ischemic Stroke

**Figure 8:**
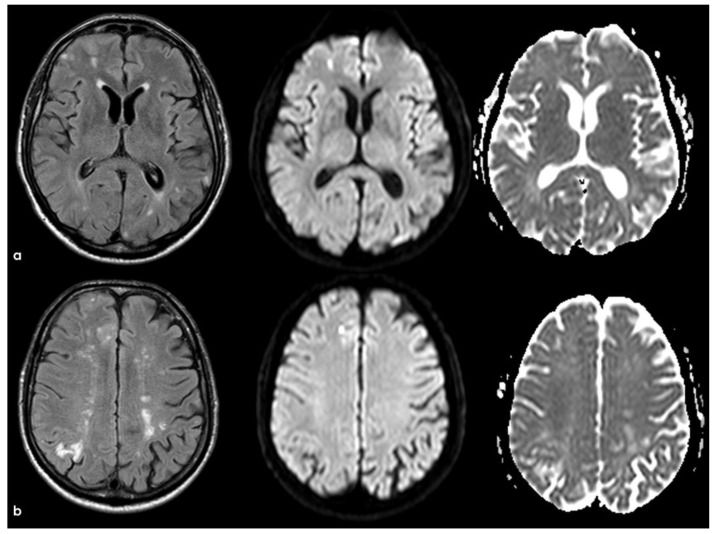
Brain MRI with axial FLAIR, DWI, and ADC slices at two different levels (panels (**a**) and (**b**)), showing two subcortical lesions in the left anterior frontal lobe, with similar signal features (hyperintense in FLAIR and DWI and hypointense on ADC map). The lesion in panel a appears less hypointense on the ADC map than the lesion in panel (**b**), probably reflecting a metachronous pattern.

The following months were uneventful, and the patient underwent a control MRI in December 2015, showing the presence of at least two left frontal subcortical FLAIT hyperintensities with DWI hyperintensity and ADC hypointensity, for subacute or recent ischemic lesions (Figure 8). Considering the underlying SVD, the previous cSAH, and the potential bleeding risk with the lack of beneficial evidence for antiplatelets, no antithrombotic therapy was added, and the reached target for vascular risk factors control was maintained.

CAA, like other SVDs, presents with both hemorrhagic and non-hemorrhagic neuroimaging features and is associated with increased ischemic and hemorrhagic risks [24,42]. The ischemic manifestations range from microinfarcts to acute DWI-positive lesions. Microinfarcts, small areas of necrosis often undetectable on conventional MRI, are more prevalent in CAA cases than non-CAA cases [43,44,45]. However, studies show inconsistent correlations between CAA severity and microinfarct burden, likely due to varied contributing factors such as arteriolosclerosis, microemboli, and hypotensive episodes [46,47]. DWI lesions, representing acute infarcts, appear in 15–23% of patients with CAA-related ICH, correlating with imaging markers like white matter hyperintensities (WMHs) and lobar cerebral microbleeds (CMBs) but not traditional vascular risk factors [47,48,49]. These lesions are linked to chronic microstructural damage and may significantly affect cognitive function [50,51,52,53,54]. A mathematical model suggests that even a few detected microinfarcts at autopsy may indicate hundreds to thousands of undetected lesions, contributing to vascular cognitive impairment in CAA [51]. Advancements in high-field MRI (3T and 7T) now enable the detection of microinfarcts as small as 1–2 mm in vivo, with ongoing improvements in T1- and T2*-weighted imaging expected to enhance the detection of even smaller lesions in the future [55,56,57,58].

## 5. February 2017: First Recurrent CAA-Related Inflammation

**Figure 9:**
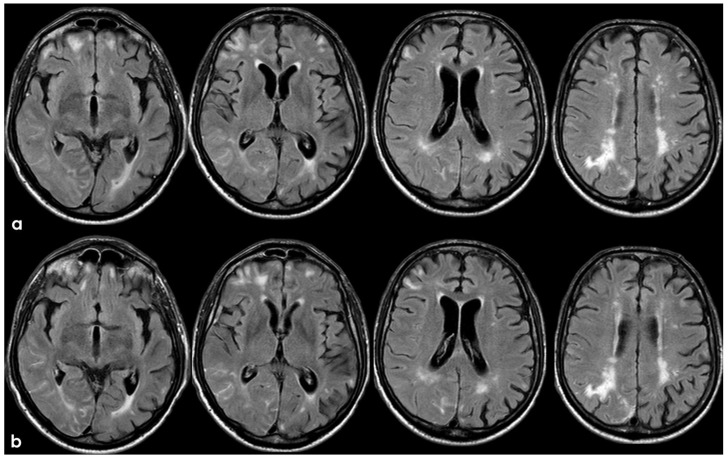
Brain MRI with axial FLAIR before (panel (**a**)) and after (panel (**b**)) contrast administration. In panel (**a**), a diffuse cortical swelling in the right temporal-occipital, frontal, and parietal lobes was evident, associated with subarachnoid hyperintensity in almost all examined sulci, and parenchymal white matter hyperintensity was evident in the right cingulus and in the right anterior frontal lobe (this was more extended than in previous MRIs). All hyperintense sulci in panel (**a**) were contrast-enhanced in panel (**b**).

The patient’s status was substantially stable in the following months, and no other events occurred until January 2017, when he complained about the subacute onset and persistence of unsteadiness, confusion, and lack of spatial orientation. The neurological exam was not informative, and a new brain MRI was planned. In February 2017, the patient underwent a brain MRI, showing a recurrence of exudative manifestations, both leptomeningeal and parenchymal ones (ARIA-E in Figure 8 and ARIA-H in Figure 9).

**Figure 10:**
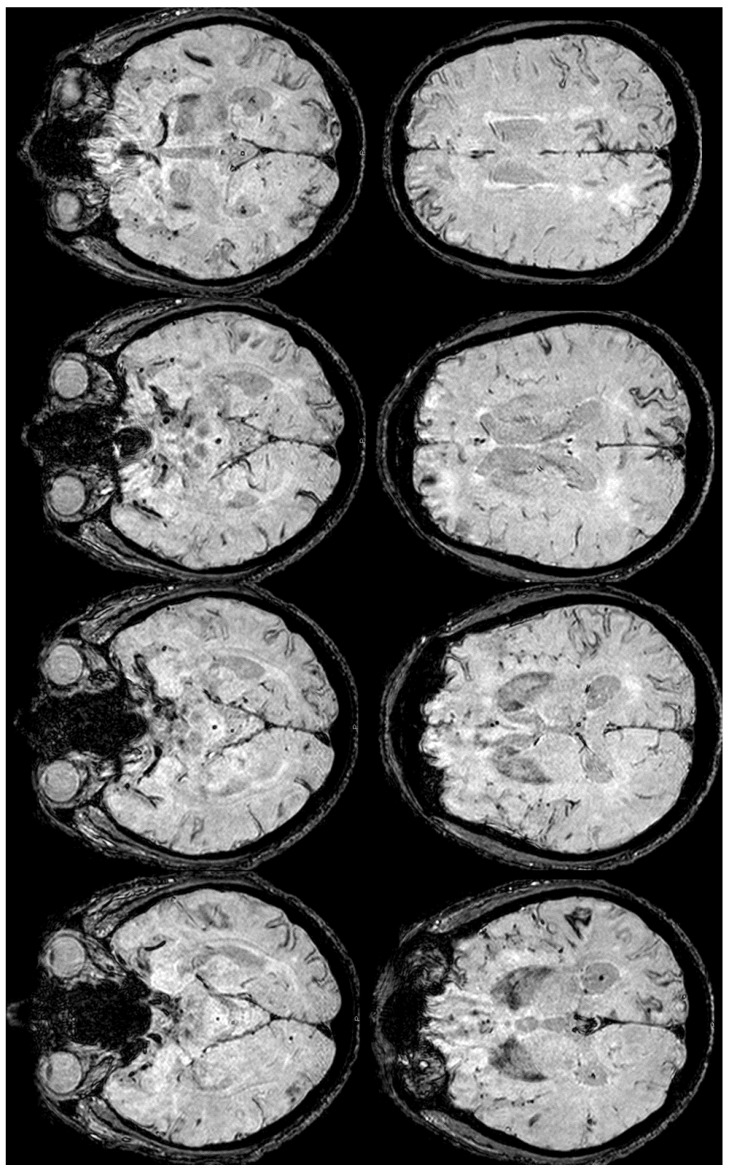
SWI-MRI in axial sections, showing an extensive, diffuse cSS, involving both occipital, temporal, and frontal lobes together with the left parietal lobe. In comparison with the previous MRI, an increased number of sulci were involved.

Meanwhile, the microhemorrhagic burden on SWI-MRI (Figure 10) was increased in extension, maintaining the preferential subarachnoid location.

**Figure 11:**
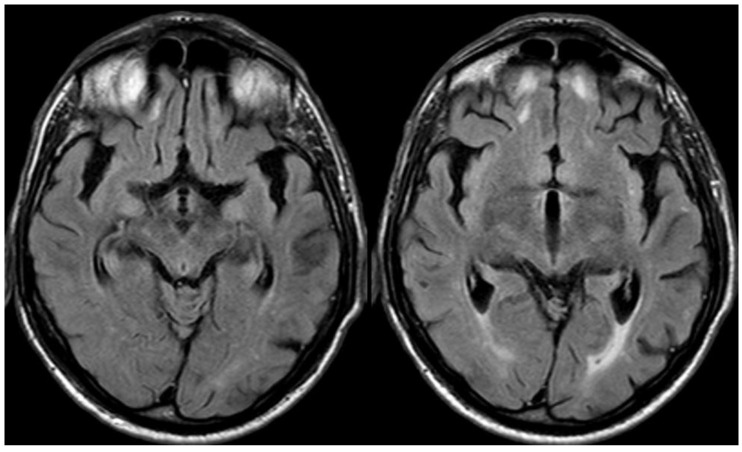
Brain MRI with unenhanced axial FALIR focused on the temporal lobes, showing the resolution of spontaneous sulcal hyperintensity and cortical swelling. The contrast administration did not find further issues.

Another time, the patient was treated using steroids, starting with Methylprednisolone, 1 gr intravenously for 5 days, and continuing with tapering off oral prednisone for eight months [23], until the neuroradiological resolution of the ARIA-E. The follow-up MRI 3 months after steroid treatment showed a strong improvement in the exudative pattern, documented by brain MRI in October 2017 (Figure 11).

**Figure 12:**
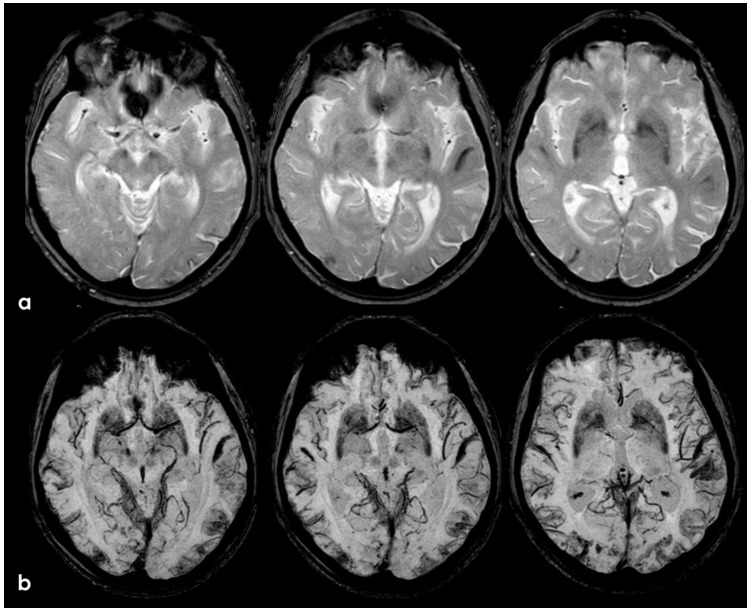
GRE and SWI-MRI (panel (**a**) and (**b**), respectively) showing the increased extent of cSS in corresponding axial slices (more evident in SWI than in GRE sequences).

At the same time point, the SWI-MRI showed a further mild extension of cSS on the right temporal sulci (Figure 12).

Initially considered a monophasic condition, CAA-related inflammation was thought to present with severe symptoms, steroid responsiveness, and no recurrences. However, early conclusions were based on case reports with limited long-term follow-up. A prospective multicenter study [23] challenged this view, revealing that relapses are more frequent than previously assumed. Defined as new symptom onset with corresponding MRI changes, relapse rates were 6.9% (95% CI 2.9–15.8%) at 3 months, 16.2% (95% CI 9.3–27.6%) at 12 months, and 38.3% (95% CI 22.9–59.2%) at 2 years. Notably, all relapsing patients had >10 cerebral microbleeds (CMBs) at baseline. Among 90 patients who achieved recovery at 3 months, 16.7% (15 patients) experienced at least one relapse during follow-up, exclusively in those with definite or probable CAA-related inflammation.

## 6. December 2017: Recurrent SAH

**Figure 13:**
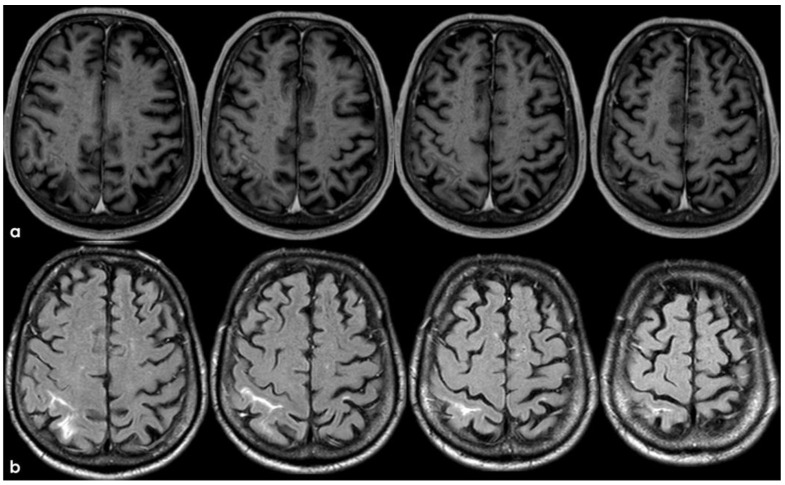
Brain MRI: post-contrast axial T1WI (panel (**a**)) and axial FLAIR (panel (**b**)), showing the contrast enhancement of the right post-central sulcus, corresponding with cSAH.

**Figure 14:**
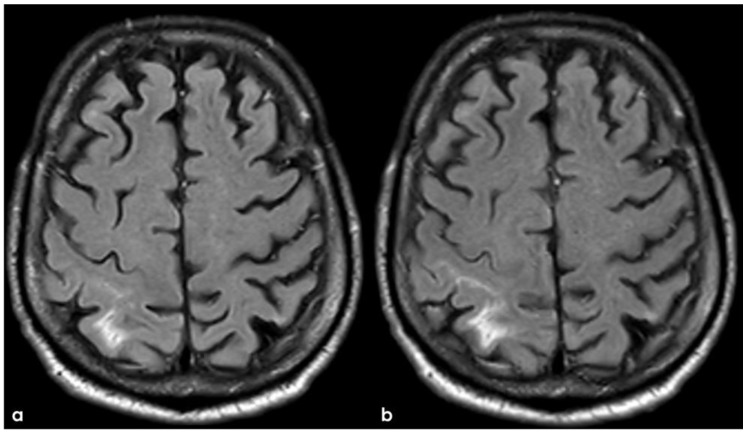
Axial FLAIR before and after contrast administration (panel (**a**) and (**b**), respectively), showing the spontaneous sulcal hyperintensity with increasing extension in a post-contrast image.

In December 2017, the patient went again to his neurologist because of the occurrence of short episodes of a lack of coordination in the left arm, lasting 1 to 5 min and having a rate of 1 to 3 times per day. Brain CT showed a recurrent cSAH in the right post-central sulcus, and the episodes were interpreted as TFNEs due to cSAH. A brain MRI was repeated (Figure 13 and Figure 14).

**Figure 15:**
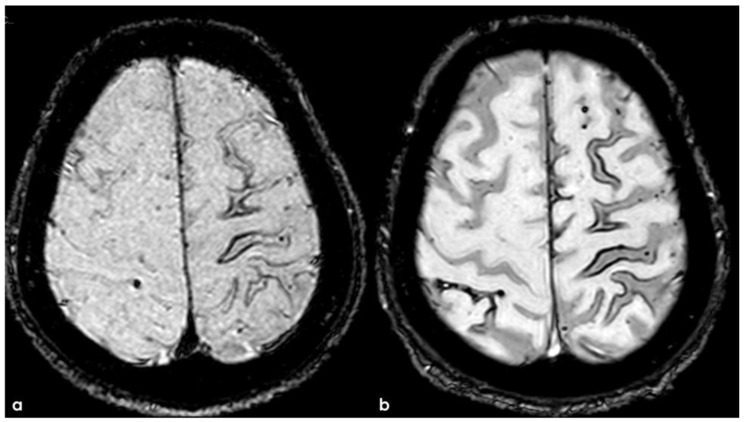
SWI MRI in 2015 (panel (**a**)) and in December 2017 (panel (**b**)), showing the evolution of sulcal involvement in the right post-central sulcus.

The corresponding SWI images were reported in Figure 15, showing a cSS in the sulcus with cSAH.

The recurrence of cSAH has been rarely addressed in the literature [32,35], and the most relevant information has been reported in Section 2. In a series of patients with cSS and/or cSAH, 29% of patients experienced a symptomatic cerebral bleeding event (either ICH or cSAH) during follow-up (average time to event: 34 months) [35]. In a recent study [32], the risk of recurrent cSAH was 11.1%/patient/year (95% CI 7.9–15.2).

## 7. April 2018: Second Recurrent CAA-Related Inflammation

**Figure 16:**
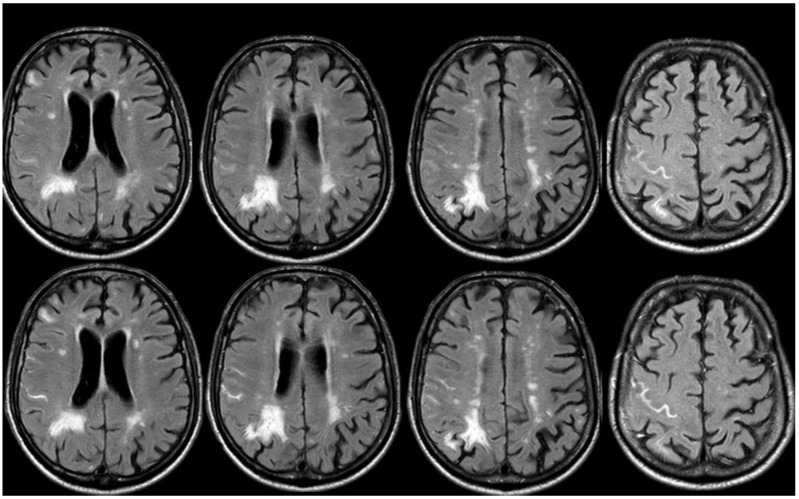
Brain MRI (axial FLAIR) before and after contrast administration (upper and lower row, respectively), showing a spontaneous hyperintense signal on several sulci in the right frontal lobe, all with mild enhancement after contrast administration and cortical ribbon swelling in the corresponding giri.

The patient experienced sporadic recurrent TFNEs with a very low rate (1/month), and in April 2018, a follow-up brain MRI was performed, showing recurrent diffuse CAA-related inflammation, mainly with a leptomeningeal pattern (Figure 16).

Steroid treatment was started, following the same schedule as the two past episodes with the early addition of non-steroid immunosuppressive treatment (azathioprine), but unfortunately, it was poorly tolerated by the patient, who underwent several severe infections at a low dosage (<1 mg/kg). These events (sepsis, recurrent urinary infections, and cholecystitis) prevent using other immunosuppressive strategies.

**Figure 17:**
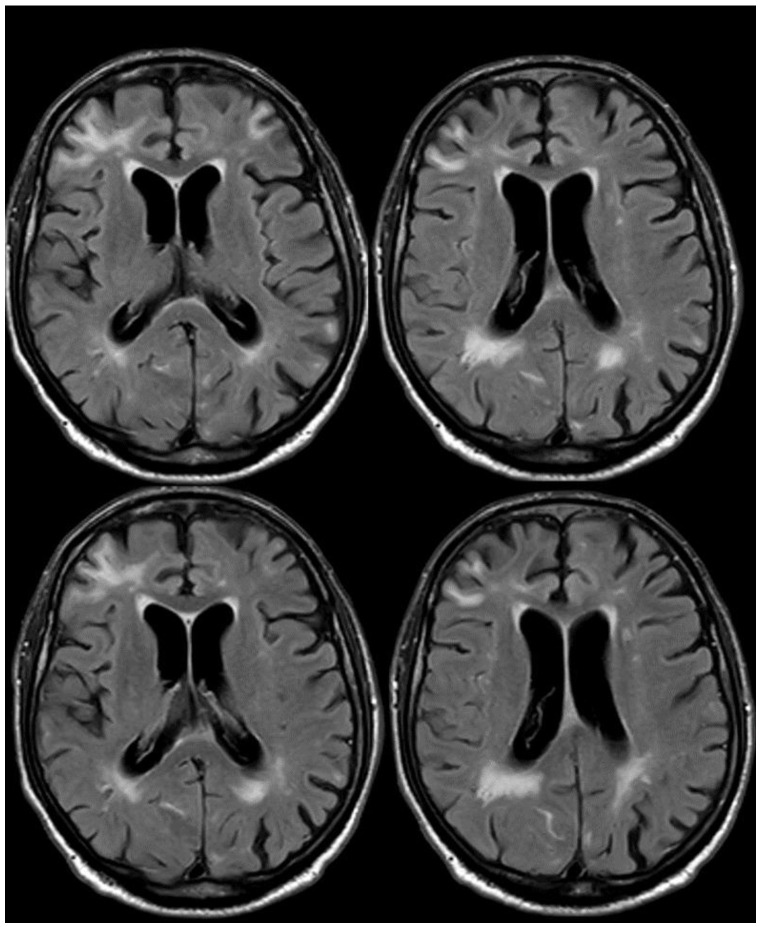
Brain MRI (axial FLAIR) before and after contrast administration (upper and lower row, respectively), showing a spontaneous hyperintense signal on the right occipital mesial lobe, all with mild enhancement after contrast administration and cortical ribbon swelling in the corresponding giri.

**Figure 18:**
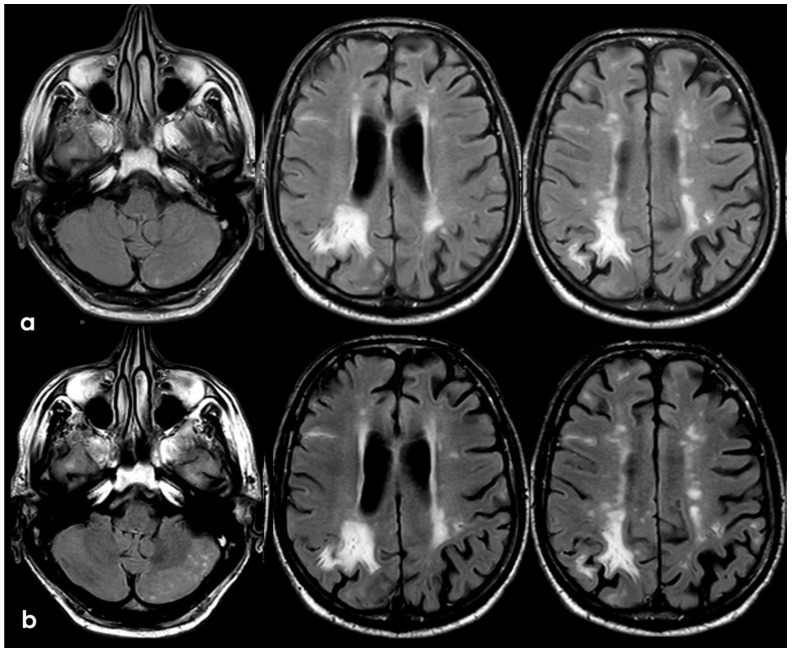
Brain MRI (axial FLAIR) before and after contrast administration (panel (**a**) and (**b**), respectively), showing a spontaneous hyperintense signal on several sulci (panel (**a**)) in the left cerebellar lobe (left column) and in the right frontal lobe (middle and right columns), all with mild enhancement after contrast administration (panel (**b**)) and cortical ribbon swelling in the corresponding giri.

The follow-up showed an almost complete resolution of the previously described sulcal FLAIR hyperintensities, except for the appearance of involvement of new sulci in July 2018 and October 2018 (Figure 17 and Figure 18, respectively).

The resolution of the exudative manifestation was documented in March 2019, while the patient maintained low-dose steroid treatment. The whole duration of the process was poorly symptomatic, with the complaint of a subjective slowing of gait and a loss of concentration but without objective deficits.

## 8. April 2018: Third Recurrent CAA-Related Inflammation

**Figure 19:**
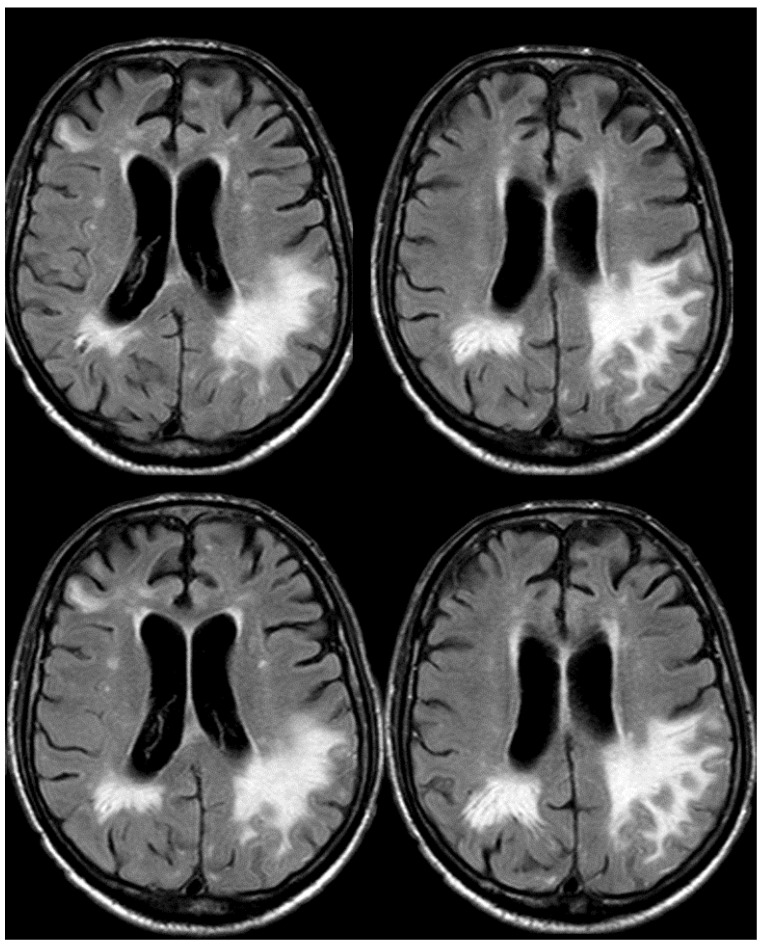
Brain MRI (axial FLAIR) before and after contrast administration (upper and lower row, respectively), showing a tumefactive white matter hyperintense signal change in the left posterior frontal and parietal lobe, sparing the U fibers and with DWI/ADC features of vasogenic edema (not shown) without contrast enhancement.

**Figure 20:**
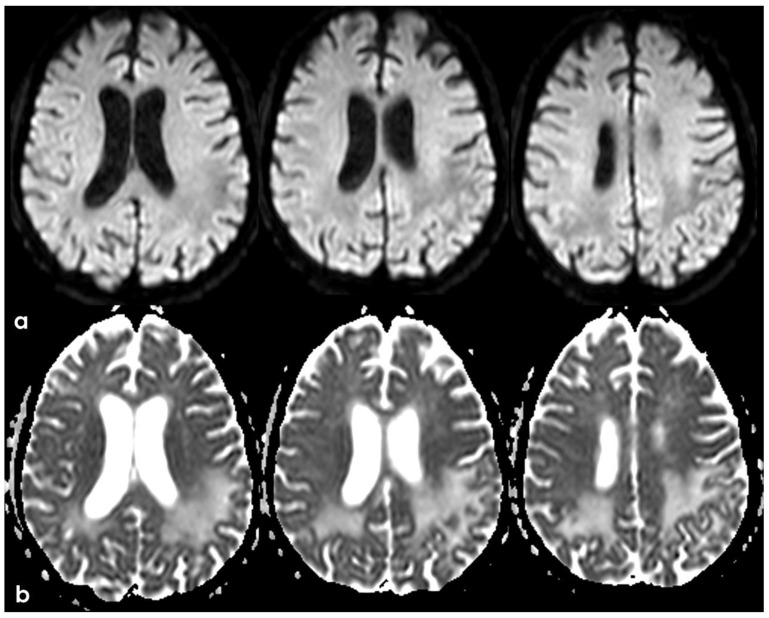
Brain MRI (axial DWI and ADC map) (panel (**a**) and (**b**), respectively), showing that the tumefactive white matter lesion shown in Figure 18 has features of vasogenic edema.

In June 2019, the patient had a focal motor seizure with access to the hospital, and after a CT scan excluded ICH, a new MRI was performed, showing classical parenchymal CAA-related inflammation involving the left posterior frontal and parietal lobes (Figure 19 and Figure 20).

**Figure 21:**
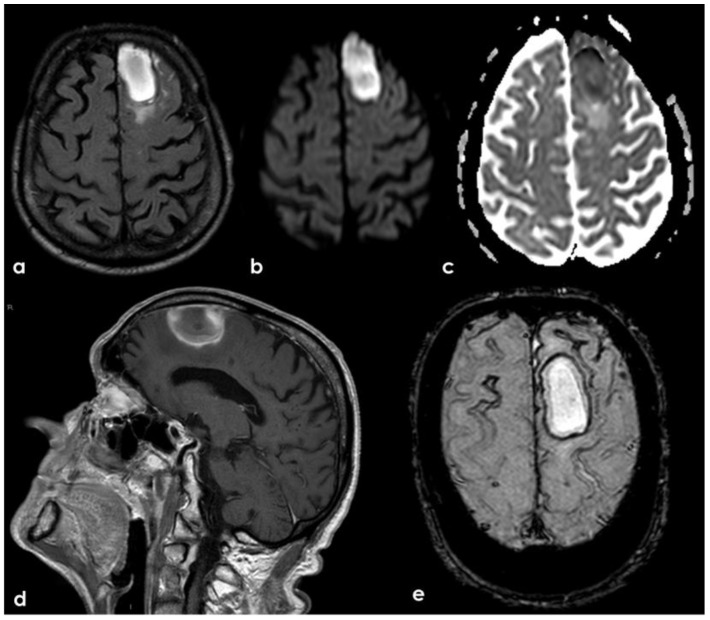
Brain MRI showing a left anterior frontal ICH on FLAIR (panel (**a**)), DWI (panel (**b**)), ADC map (panel (**c**)), coronal T1WI (panel (**d**)), and SWI (panel (**e**)).

A new IV steroid pulse was administered with quick improvement, together with antiepileptic treatment. In September 2019, a few days before the planned control MRI, the patient experienced a mild slowing of speech and a reduction in motor initiative. The control MRI showed the resolution of the previously presented CAA-related inflammation and a left frontal ICH (Figure 21).

The CAA-related ICH risk is well known, particularly in patients with disseminated cSS, being approximately three times the hazard ratio for recurrent hemorrhage than those without cSS [59,60,61]. In addition, cSS was associated with a poor functional prognosis in patients with CAA with or without an ICH or cSAH history [62].

The evolution was a slow-progressing cognitive impairment with a lack of motor performance and a bed-ridden status until death, occurring after 1 year because of ab ingestis pneumonia.

## 9. Additional Information and Discussion: How This Case Helps to Better Understand CAA

The case presented has some unique characteristics worth considering. Initially, the diagnosis of CAA was made using the Boston 1.0 [63] or Boston 1.5 criteria [64]. Given that the patient had a single isolated lobar microbleed (MB) and did not meet the criteria for cSS (under Boston 1.0), they were classified as “possible CAA” due to the absence of ICH. However, the updated Boston 2.0 criteria, which now include cSAH among hemorrhagic events alongside ICH, would have classified the patient as “probable CAA” from the outset [24]. This change increases the diagnostic weight of cSAH and TFNEs, both of which are highly indicative of CAA. The presence of non-hemorrhagic MRI markers such as white matter hyperintensities (WMHs) and enlarged perivascular spaces further supports the diagnosis, as these markers are incorporated into the Boston 2.0 criteria [24]. Under the Boston 1.5 criteria, which include cSS, the patient would still be considered “possible CAA” without a history of ICH [64,65]. In addition to the existing diagnostic criteria, other tools and techniques have provided a higher probability definition of CAA. However, the performance of the Boston 2.0 criteria in patients without ICH has been recently defined [31,66,67]. The patient’s ApoE genotype (E3/E3), not strongly associated with brain bleeding risk, could have played a role in the long period before the patient experienced ICH, despite having disseminated and multifocal cSS and at least two episodes of cSAH. Initially, evaluations of cSS as a bleeding risk driver focused on patients with a history of ICH, not those with possible CAA diagnosed by the earlier Boston criteria [64,65]. This patient selection may have caused an imbalance in the ApoE genotypes, leading to an overrepresentation of genotypes associated with higher bleeding risk, such as E2 and E4. A multicenter prospective study evaluating the prognostic role of cSS, independent of whether the patient had possible or probable CAA, showed that cSS—particularly disseminated—was associated with significantly worse functional prognosis and higher mortality within 12 months of follow-up [62]. Patients with cSS had a higher incidence of both ICH (17% vs. 4%, *p* = 0.0003) and functional dependence (59% vs. 82%, *p* = 0.00002). The presence of cSS was independently associated with incident ICH and poorer outcomes, as measured by the modified Rankin Scale (mRS) [62]. The mechanism linking cSS to an increased risk of ICH remains unclear. One hypothesis proposes that increased prevalence of the APOE ε2 allele among those with cSS may contribute to amyloid-related vascular degenerative changes that increase bleeding risk [65,68,69,70]. Another hypothesis suggests a direct toxic effect of superficial blood on the underlying cortex, possibly triggering spreading depolarization in cortical neurons, which may be linked to TFNEs [71,72,73]. However, it is uncertain whether this process increases neuronal injury or the risk of subsequent bleeding [74,75]. TFNEs are considered characteristic of CAA, especially in the context of cSS and cSAH, as they have not been observed in patients with SVDs other than CAA or in patients with different causes of cSAH and cSS [27]. Furthermore, given the frequent comorbidity between CAA and Alzheimer’s disease (AD), TFNEs are not surprising in AD patients and have been described as TIA-like episodes [76]. In a systematic review and meta-analysis of 222 CAA-associated TFNE cases, cSAH or cSS was detected in 77.8% of patients, and symptomatic lobar ICH occurred in 39.4% during follow-up. Lobar ICH and cSS were identified as key risk factors for death (odds ratio [OR], 3.01 [95% CI, 1.36–6.69]; OR, 3.20 [95% CI, 1.16–8.91], respectively) [77].

Finally, CAA-related inflammation and amyloid-related imaging abnormalities (ARIAs) in AD share similar clinical manifestations and neuroradiological patterns, suggesting a potential overlap. In the case presented, neuroradiological evidence of leptomeningeal involvement was observed in most episodes, which has been reported in isolation in patients with ARIAs [25]. The association between CAA-related inflammation and ApoE genotypes (E2 or E4), noted in earlier reports, may not be as strong in large prospective case series [23]. Studies show that over 30% of patients with CAA had the E3/E3 genotype, indicating that the ApoE genotype may not be as predictive of CAA outcomes as previously thought. Immunotherapy studies on AD, which focus on patients with early AD and the E4 genotype, may also suffer from patient selection bias, limiting the generalizability of these findings [15,36].

Other elements that can be useful in an ancillary manner with respect to the diagnostic criteria of CAA are precisely the liquor biomarkers of neurodegeneration, acquired several times in the case presented, but substantially interlocutory in the results when acquired in the clinical and neuroradiological quiescence phase of the disease [78]. Conversely, the functional data of the PET with Flumetamol in the patient we presented, performed before the last hemorrhagic event, showed a widespread and almost ubiquitous uptake of the radiotracer at the cortical level, confirming, although without specificity for vascular forms, the presence of an amyloid-related disease in the brain [79,80].

CAA-related inflammation is a broader disease category than amyloid-beta-related angiitis (ABRA), a topic still debated in the literature [81,82]. ABRA is histopathologically defined by the presence of amyloid-beta fragments and transmural inflammation, typically accompanied by microhemorrhagic markers on MRI. However, unlike CAA, which is linked to small vessel disease (SVD), ABRA is not strictly associated with ICH. A study by Scolding et al. [82] found that only 1 of 9 patients with ABRA experienced ICH, and it did not occur at disease onset. In contrast, all patients showed tumefactive white matter involvement, often affecting multiple lobes, resembling the neuroimaging features of common CAA-related inflammation [26]. In the largest cohort study of CAA-related inflammation, 33.6% of patients had a history of ICH at enrollment, and 71.7% were classified as probable CAA according to the Boston criteria, with an odds ratio (OR) for developing ICH within 24 months at 17.1 (95% CI 8.5–32.7) [23]. These findings suggest that ABRA could represent a distinct disease, with beta-amyloid possibly acting as a secondary or incidental factor in the disease process.

Distinguishing ABRA from CAA-related inflammation is challenging because ABRA relies on histopathological diagnosis, as required for all subtypes of primary angiitis of the central nervous system (PACNS). Moreover, ABRA has a relatively benign course, shares clinical and neuroimaging features with CAA-related inflammation (which has validated clinical and neuroradiological criteria), and overlaps in therapeutic approaches, making it harder to describe ABRA patient cohorts reliably. Histopathologically, the primary difference between these conditions in the acute phase lies in the localization of the inflammatory infiltrate: perivascular in CAA-related inflammation and transmural in ABRA. Additionally, Alzheimer’s disease-associated histopathological features are less common and less significant in ABRA but more frequent in CAA-related inflammation [83,84]. A recent French cohort study on PACNS [83] emphasized differences between CAA-related inflammation and biopsy-proven PACNS. Specific markers for CAA-related inflammation included hemorrhagic subarachnoid involvement, a history of ICH, ≥21 visible centrum semiovale perivascular spaces, and fulfillment of probable CAA-related inflammation criteria. Recent studies [72,73,84,85] have confirmed the existence of a leptomeningeal variant of CAA-related inflammation, which, as seen in the presented case, is more commonly associated with TFNEs than with parenchymal involvement. This variant closely resembles the leptomeningeal pattern observed in amyloid-related imaging abnormalities (ARIAs). Inflammatory mechanisms have been suggested for the genesis of microbleeds too, and this makes inflammatory CAA a putative mechanism for the MRI hallmark of the disease [86,87,88,89].

Finally, recent research on hereditary, sporadic, and iatrogenic CAA outlined a framework describing disease progression over approximately three decades [90]. The progression typically unfolds across four distinct stages: (I) initial Aβ deposition in small arterioles and capillaries; (II) deterioration of vascular physiological reactivity, leading to impaired blood vessel function; (III) emergence of non-hemorrhagic tissue damage, including ischemic lesions and white matter hyperintensities; (IV) breakdown of the vessel wall and hemorrhage, marking the advanced phase of the disease. Postmortem studies further reveal that, in the final stage, vascular remodeling occurs, potentially triggered by immune-mediated clearance of Aβ deposits [20,91,92,93,94,95,96,97]. This mechanism may underlie some of the hemorrhagic events and it may be involved in CAA-related inflammation and/or ARIA development. A pilot investigation, using tracers for activated glial cells, demonstrated a persisting capitation in the brain outside the areas involved in CAA-related inflammation, suggesting a role for a baseline inflammatory mechanism in the pathophysiology of CAA manifestations and in the progression of neuroradiological markers over time [20].

This study has certain limitations, particularly due to its reliance on an individual case description without the inclusion of a control cohort. This restriction could impact the generalizability of the findings to a larger population of CAA patients. Nevertheless, the study has notable strengths, especially in demonstrating how some patients may experience a prolonged disease course involving nearly all potential clinical and neuroradiological manifestations of CAA, including TFNEs, cSAH, CAA-related inflammation, ischemic stroke, and ICH. The patient in this study experienced four distinct episodes of CAA-related inflammation, with three showing exclusive or predominant leptomeningeal involvement and one with parenchymal involvement. This highlights the complexity of the disease’s natural history, which is often overlooked in earlier series, mainly due to their retrospective design and shorter follow-up periods. However, this pattern has been confirmed in a larger prospective cohort of CAA-related inflammation patients.

## 10. Conclusions

The presented case shows how, even in diseases with a marked connotation, the individual trajectory of the disease can be long and characterized by multiple steps as clinical and neuroradiological manifestations. All this contributes to defining a burden of microangiopathic damage that increases as a temporal (and perhaps etiological) consequence of these manifestations. It is possible that the new diagnostic criteria of CAA can focus attention on clinical presentations different from lobar ICH and help to identify different phenotypes of the disease and to better understand the physiopathology of the underlying SVD. In the meantime, the knowledge and identification of different clinical and neuroradiological phenotypes of CAA may help in designing disease trajectories to act on in an individualized manner, considering CAA-related inflammation as a target for future therapeutic trials.

## Data Availability

No new data were created or analyzed in this study. Data sharing is not applicable to this article.

## References

[1] Jokinen H., Koikkalainen J., Laakso H.M., Melkas S., Nieminen T., Brander A., Korvenoja A., Rueckert D., Barkhof F., Scheltens P. (2020). Global Burden of Small Vessel Disease-Related Brain Changes on MRI Predicts Cognitive and Functional Decline. Stroke.

[2] Jansma A., de Bresser J., Schoones J.W., van Heemst D., Akintola A.A. (2024). Sporadic cerebral small vessel disease and cognitive decline in healthy older adults: A systematic review and meta-analysis. J. Cereb. Blood Flow Metab..

[3] Seiffge D.J., Wilson D., Ambler G., Banerjee G., Hostettler I.C., Houlden H., Shakeshaft C., Cohen H., Yousry T.A., Salman R.A.-S. (2021). Small vessel disease burden and intracerebral haemorrhage in patients taking oral anticoagulants. J. Neurol. Neurosurg. Psychiatry.

[4] Wardlaw J.M., Smith C., Dichgans M. (2019). Small vessel disease: Mechanisms and clinical implications. Lancet Neurol..

[5] Mahammedi A., Wang L., Williamson B., Khatri P., Kissela B., Sawyer R., Shatz R., Khandwala V., Vagal A. (2022). Small Vessel Disease, a Marker of Brain Health: What the Radiologist Needs to Know. AJNR Am. J. Neuroradiol..

[6] Duering M., Biessels G.J., Brodtmann A., Chen C., Cordonnier C., de Leeuw F.-E., Debette S., Frayne R., Jouvent E., Rost N.S. (2023). Neuroimaging standards for research into small vessel disease-advances since 2013. Lancet Neurol..

[7] Puy L., Pasi M., Rodrigues M., van Veluw S.J., Tsivgoulis G., Shoamanesh A., Cordonnier C. (2021). Cerebral microbleeds: From depiction to interpretation. J. Neurol. Neurosurg. Psychiatry.

[8] Pasi M., Charidimou A., Boulouis G., Auriel E., Ayres A., Schwab K.M., Goldstein J.N., Rosand J., Viswanathan A., Pantoni L. (2018). Mixed-location cerebral hemorrhage/microbleeds: Underlying microangiopathy and recurrence risk. Neurology.

[9] Charidimou A., Linn J., Vernooij M.W., Opherk C., Akoudad S., Baron J.-C., Greenberg S.M., Jäger H.R., Werring D.J. (2015). Cortical superficial siderosis: Detection and clinical significance in cerebral amyloid angiopathy and related conditions. Brain.

[10] Greenberg S.M., Bacskai B.J., Hernandez-Guillamon M., Pruzin J., Sperling R., van Veluw S.J. (2020). Cerebral amyloid angiopathy and Alzheimer disease—One peptide, two pathways. Nat. Rev. Neurol..

[11] Piazza F., Winblad B. (2016). Amyloid-Related Imaging Abnormalities (ARIA) in Immunotherapy Trials for Alzheimer’s Disease: Need for Prognostic Biomarkers?. J. Alzheimers Dis..

[12] Loeffler D.A. (2023). Antibody-Mediated Clearance of Brain Amyloid-β: Mechanisms of Action, Effects of Natural and Monoclonal Anti-Aβ Antibodies, and Downstream Effects. J. Alzheimers Dis. Rep..

[13] Sperling R.A., Jack C.R., Black S.E., Frosch M.P., Greenberg S.M., Hyman B.T., Scheltens P., Carrillo M.C., Thies W., Bednar M.M. (2011). Amyloid-related imaging abnormalities in amyloid-modifying therapeutic trials: Recommendations from the Alzheimer’s Association Research Roundtable Workgroup. Alzheimers Dement..

[14] Agarwal A., Gupta V., Brahmbhatt P., Desai A., Vibhute P., Joseph-Mathurin N., Bathla G. (2023). Amyloid-related Imaging Abnormalities in Alzheimer Disease Treated with Anti-Amyloid-β Therapy. Radiographics.

[15] Barakos J., Purcell D., Suhy J., Chalkias S., Burkett P., Grassi C.M., Castrillo-Viguera C., Rubino I., Vijverberg E. (2022). Detection and Management of Amyloid-Related Imaging Abnormalities in Patients with Alzheimer’s Disease Treated with Anti-Amyloid Beta Therapy. J. Prev. Alzheimers Dis..

[16] Roytman M., Mashriqi F., Al-Tawil K., Schulz P.E., Zaharchuk G., Benzinger T.L.S., Franceschi A.M. (2023). Amyloid-Related Imaging Abnormalities: An Update. AJR Am. J. Roentgenol..

[17] Jeong S.Y., Suh C.H., Shim W.H., Lim J.-S., Lee J.-H., Kim S.J. (2022). Incidence of Amyloid-Related Imaging Abnormalities in Patients With Alzheimer Disease Treated With Anti-β-Amyloid Immunotherapy: A Meta-analysis. Neurology.

[18] Hampel H., Elhage A., Cho M., Apostolova L.G., Nicoll J.A.R., Atri A. (2023). Amyloid-related imaging abnormalities (ARIA): Radiological, biological and clinical characteristics. Brain.

[19] DiFrancesco J.C., Longoni M., Piazza F. (2015). Anti-Aβ Autoantibodies in Amyloid Related Imaging Abnormalities (ARIA): Candidate Biomarker for Immunotherapy in Alzheimer’s Disease and Cerebral Amyloid Angiopathy. Front. Neurol..

[20] Piazza F., Caminiti S.P., Zedde M., Presotto L., DiFrancesco J.C., Pascarella R., Giossi A., Sessa M., Poli L., Basso G. (2022). Association of Microglial Activation With Spontaneous ARIA-E and CSF Levels of Anti-Aβ Autoantibodies. Neurology.

[21] Piazza F., Greenberg S.M., Savoiardo M., Gardinetti M., Chiapparini L., Raicher I., Nitrini R., Sakaguchi H., Brioschi M., Billo G. (2013). Anti-amyloid β autoantibodies in cerebral amyloid angiopathy-related inflammation: Implications for amyloid-modifying therapies. Ann. Neurol..

[22] Zedde M., Pascarella R., Piazza F. (2023). CAA-ri and ARIA: Two Faces of the Same Coin?. AJNR Am. J. Neuroradiol..

[23] Antolini L., DiFrancesco J.C., Zedde M., Basso G., Arighi A., Shima A., Cagnin A., Caulo M., Carare R.O., Charidimou A. (2021). Spontaneous ARIA-like Events in Cerebral Amyloid Angiopathy-Related Inflammation: A Multicenter Prospective Longitudinal Cohort Study. Neurology.

[24] Charidimou A., Boulouis G., Frosch M.P., Baron J.-C., Pasi M., Albucher J.F., Banerjee G., Barbato C., Bonneville F., Brandner S. (2022). The Boston criteria version 2.0 for cerebral amyloid angiopathy: A multicentre, retrospective, MRI-neuropathology diagnostic accuracy study. Lancet Neurol..

[25] Cogswell P., Barakos J., Barkhof F., Benzinger T., Jack C., Poussaint T., Raji C., Ramanan V., Whitlow C. (2022). Amyloid-Related Imaging Abnormalities with Emerging Alzheimer Disease Therapeutics: Detection and Reporting Recommendations for Clinical Practice. AJNR Am. J. Neuroradiol..

[26] Auriel E., Charidimou A., Gurol M.E., Ni J., Van Etten E.S., Martinez-Ramirez S., Boulouis G., Piazza F., DiFrancesco J.C., Frosch M.P. (2016). Validation of Clinicoradiological Criteria for the Diagnosis of Cerebral Amyloid Angiopathy-Related Inflammation. JAMA Neurol..

[27] Zedde M., Grisendi I., Assenza F., Napoli M., Moratti C., Pavone C., Bonacini L., Di Cecco G., D’aniello S., Pezzella F.R. (2024). Spontaneous Non-Aneurysmal Convexity Subarachnoid Hemorrhage: A Scoping Review of Different Etiologies beyond Cerebral Amyloid Angiopathy. J. Clin. Med..

[28] Khurram A., Kleinig T., Leyden J. (2014). Clinical associations and causes of convexity subarachnoid hemorrhage. Stroke.

[29] Kumar S., Goddeau R.P., Selim M.H., Thomas A., Schlaug G., Alhazzani A., Searls D.E., Caplan L.R. (2010). Atraumatic convexal subarachnoid hemorrhage: Clinical presentation, imaging patterns, and etiologies. Neurology.

[30] Greenberg S.M., Vonsattel J., Stakes J.W., Gruber M., Finklestein S.P. (1993). The clinical spectrum of cerebral amyloid angiopathy: Presentations without lobar hemorrhage. Neurology.

[31] Switzer A.R., Charidimou A., McCarter S., Vemuri P., Nguyen A.T., Przybelski S.A., Lesnick T.G., Rabinstein A.A., Brown R.D., Knopman D.S. (2024). Boston Criteria v2.0 for Cerebral Amyloid Angiopathy Without Hemorrhage: An MRI-Neuropathologic Validation Study. Neurology.

[32] Hostettler I.C., Wilson D., Fiebelkorn C.A., Aum D., Ameriso S.F., Eberbach F., Beitzke M., Kleinig T., Phan T., Marchina S. (2022). Risk of intracranial haemorrhage and ischaemic stroke after convexity subarachnoid haemorrhage in cerebral amyloid angiopathy: International individual patient data pooled analysis. J. Neurol..

[33] Regenhardt R.W., Thon J.M., Das A.S., Thon O.R., Charidimou A., Viswanathan A., Gurol M.E., Chwalisz B.K., Frosch M.P., Cho T.A. (2020). Association between immunosuppressive treatment and outcomes of cerebral amyloid angiopathy-related inflammation. JAMA Neurol..

[34] Smith E.E., Charidimou A., Ayata C., Werring D.J., Greenberg S.M. (2021). Cerebral Amyloid Angiopathy-Related Transient Focal Neurologic Episodes. Neurology.

[35] Ni J., Auriel E., Jindal J., Ayres A., Schwab K.M., Martinez-Ramirez S., Gurol E.M., Greenberg S.M., Viswanathan A. (2015). The characteristics of superficial siderosis and convexity subarachnoid hemorrhage and clinical relevance in suspected cerebral amyloid angiopathy. Cerebrovasc. Dis..

[36] Sperling R., Salloway S., Brooks D.J., Tampieri D., Barakos J., Fox N.C., Raskind M., Sabbagh M., Honig L.S., Porsteinsson A.P. (2012). Amyloid-related imaging abnormalities in patients with Alzheimer’s disease treated with bapineuzumab: A retrospective analysis. Lancet Neurol..

[37] Filippi M., Cecchetti G., Spinelli E.G., Vezzulli P., Falini A., Agosta F. (2022). Amyloid-Related Imaging Abnormalities and β-Amyloid-Targeting Antibodies: A Systematic Review. JAMA Neurol..

[38] Werring D.J., Sperling R. (2013). Inflammatory cerebral amyloid angiopathy and amyloid-modifying therapies: Variations on the same ARIA?. Ann. Neurol..

[39] Cogswell P.M., Andrews T.J., Barakos J.A., Barkhof F., Bash S., Benayoun M.D., Chiang G.C., Franceschi A.M., Jack C.R., Pillai J.J. (2024). Alzheimer’s Disease Anti-Amyloid Immunotherapies: Imaging Recommendations and Practice Considerations for ARIA Monitoring. AJNR Am. J. Neuroradiol..

[40] Sin M.-K., Zamrini E., Ahmed A., Nho K., Hajjar I. (2023). Anti-Amyloid Therapy, AD, and ARIA: Untangling the Role of CAA. J. Clin. Med..

[41] https://sites.google.com/site/icabinternationalnetwork/home.

[42] Reijmer Y.D., van Veluw S.J., Greenberg S.M. (2016). Ischemic brain injury in cerebral amyloid angiopathy. J. Cereb. Blood Flow Metab..

[43] Smith E.E., Schneider J.A., Wardlaw J.M., Greenberg S.M. (2012). Cerebral microinfarcts: The invisible lesions. Lancet Neurol..

[44] Brundel M., de Bresser J., van Dillen J.J., Kappelle L.J., Biessels G.J. (2012). Cerebral microinfarcts: A systematic review of neuropathological studies. J. Cereb. Blood Flow Metab..

[45] Okamoto Y., Yamamoto T., Kalaria R.N., Senzaki H., Maki T., Hase Y., Kitamura A., Washida K., Yamada M., Ito H. (2012). Cerebral hypoperfusion accelerates cerebral amyloid angiopathy and promotes cortical microinfarcts. Acta Neuropathol..

[46] Launer L.J., Petrovitch H., Ross G.W., Markesbery W., White L.R. (2008). AD brain pathology: Vascular origins? Results from the HAAS autopsy study. Neurobiol. Aging.

[47] Gregoire S.M., Charidimou A., Gadapa N., Dolan E., Antoun N., Peeters A., Vandermeeren Y., Laloux P., Baron J.-C., Jäger H.R. (2011). Acute ischaemic brain lesions in intracerebral haemorrhage: Multicentre cross-sectional magnetic resonance imaging study. Brain.

[48] Kimberly W.T., Gilson A., Rost N.S., Rosand J., Viswanathan A., Smith E.E., Greenberg S.M. (2009). Silent ischemic infarcts are associated with hemorrhage burden in cerebral amyloid angiopathy. Neurology.

[49] Auriel E., Gurol M.E., Ayres A., Dumas A.P., Schwab K.M., Vashkevich A., Martinez-Ramirez S., Rosand J., Viswanathan A., Greenberg S.M. (2012). Characteristic distributions of intracerebral hemorrhage-associated diffusion-weighted lesions. Neurology.

[50] Auriel E., Edlow B.L., Reijmer Y.D., Fotiadis P., Ramirez-Martinez S., Ni J., Reed A.K., Vashkevich A., Schwab K., Rosand J. (2014). Microinfarct disruption of white matter structure: A longitudinal diffusion tensor analysis. Neurology.

[51] Westover M.B., Bianchi M.T., Yang C., Schneider J.A., Greenberg S.M. (2013). Estimating cerebral microinfarct burden from autopsy samples. Neurology.

[52] Sonnen J.A., Larson E.B., Crane P.K., Haneuse S., Li G., Schellenberg G.D., Craft S., Leverenz J.B., Montine T.J. (2007). Pathological correlates of dementia in a longitudinal, population-based sample of aging. Ann. Neurol..

[53] Launer L.J., Hughes T.M., White L.R. (2011). Microinfarcts brain atrophy, and cognitive function: The Honolulu Asia Aging Study Autopsy Study. Ann. Neurol..

[54] Sinka L., Kövari E., Gold G., Hof P.R., Herrmann F.R., Bouras C., Giannakopoulos P. (2010). Small vascular and Alzheimer disease-related pathologic determinants of dementia in the oldest old. J. Neuropathol. Exp. Neurol..

[55] van Veluw S.J., Zwanenburg J.J., Engelen-Lee J., Spliet W.G., Hendrikse J., Luijten P.R., Biessels G.J. (2013). In vivo detection of cerebral cortical microinfarcts with high-resolution 7T MRI. J. Cereb. Blood Flow Metab..

[56] van Veluw S.J., Zwanenburg J.J., Rozemuller A.J., Luijten P.R., Spliet W.G., Biessels G.J. (2015). The spectrum of MR detectable cortical microinfarcts: A classification study with 7-tesla postmortem MRI and histopathology. J. Cereb. Blood Flow Metab..

[57] Van Rooden S., Doan N.T., Versluis M.J., Goos J.D., Webb A.G., Oleksik A.M., van der Flier W.M., Scheltens P., Barkhof F., Weverling–Rynsburger A.W. (2015). 7T T2(*)-weighted magnetic resonance imaging reveals cortical phase differences between early- and late-onset Alzheimer’s disease. Neurobiol. Aging.

[58] Salat D., Chen J., van der Kouwe A., Greve D., Fischl B., Rosas H. (2011). Hippocampal degeneration is associated with temporal and limbic gray matter/white matter tissue contrast in Alzheimer’s disease. Neuroimage.

[59] Charidimou A., Peeters A.P., Jäger R., Fox Z., Vandermeeren Y., Laloux P., Baron J.-C., Werring D.J. (2013). Cortical superficial siderosis and intracerebral hemorrhage risk in cerebral amyloid angiopathy. Neurology.

[60] Charidimou A., Boulouis G., Roongpiboonsopit D., Auriel E., Pasi M., Haley K., van Etten E.S., Martinez-Ramirez S., Ayres A., Vashkevich A. (2017). Cortical superficial siderosis multifocality in cerebral amyloid angiopathy: A prospective study. Neurology.

[61] Moulin S., Casolla B., Kuchcinski G., Boulouis G., Rossi C., Henon H., Leys D., Cordonnier C. (2018). Cortical superficial siderosis: A prospective observational cohort study. Neurology.

[62] Wollenweber F.A., Opherk C., Zedde M., Catak C., Malik R., Duering M., Konieczny M.J., Pascarella R., Samões R., Correia M. (2019). Prognostic relevance of cortical superficial siderosis in cerebral amyloid angiopathy. Neurology.

[63] Knudsen K.A., Rosand J., Karluk D., Greenberg S.M. (2001). Clinical diagnosis of cerebral amyloid angiopathy: Validation of the Boston criteria. Neurology.

[64] Charidimou A., Boulouis G. (2022). Clinical Diagnosis of Probable Cerebral Amyloid Angiopathy: Diagnostic Accuracy Meta-Analysis of the Boston Criteria. Stroke.

[65] Charidimou A., Jäger R.H., Fox Z., Peeters A., Vandermeeren Y., Laloux P., Baron J.-C., Werring D.J. (2013). Prevalence and mechanisms of cortical superficial siderosis in cerebral amyloid angiopathy. Neurology.

[66] Fandler-Höfler S., Gattringer T., Enzinger C., Werring D.J. (2023). Comparison of Boston Criteria v2.0/v1.5 for Cerebral Amyloid Angiopathy to Predict Recurrent Intracerebral Hemorrhage. Stroke.

[67] Raposo N., Périole C., Planton M. (2024). In-vivo diagnosis of cerebral amyloid angiopathy: An updated review. Curr. Opin. Neurol..

[68] Roongpiboonsopit D., Charidimou A., William C.M., Lauer A., Falcone G.J., Martinez-Ramirez S., Biffi A., Ayres A., Vashkevich A., Awosika O.O. (2016). Cortical superficial siderosis predicts early recurrent lobar hemorrhage. Neurology.

[69] Pichler M., Vemuri P., Rabinstein A.A., Aakre J., Flemming K.D., Brown R.D., Kumar N., Kantarci K., Kremers W., Mielke M.M. (2017). Prevalence and natural history of superficial siderosis: A population-based study. Stroke.

[70] Mandybur T.I. (1986). Cerebral amyloid angiopathy: The vascular pathology and complications. J. Neuropathol. Exp. Neurol..

[71] Chung D.Y., Oka F., Ayata C. (2016). Spreading depolarizations: A therapeutic target against delayed cerebral ischemia after subarachnoid hemorrhage. J. Clin. Neurophysiol..

[72] Charidimou A. (2025). Cerebral Amyloid Angiopathy-Related Inflammation Spectrum Disorders: Introduction of a Novel Concept and Diagnostic Criteria. Ann. Neurol..

[73] Sellimi A., Panteleienko L., Mallon D., Fandler-Höfler S., Oliver R., Harvey V., Zandi M.S., Banerjee G., Werring D.J. (2025). Inflammation in Cerebral Amyloid Angiopathy-Related Transient Focal Neurological Episodes. Ann. Neurol..

[74] Charidimou A., Boulouis G., Xiong L., Pasi M., Roongpiboonsopit D., Ayres A., Schwab K.M., Rosand J., Gurol M.E., Viswanathan A. (2019). Cortical Superficial Siderosis Evolution. Stroke.

[75] Ringman J.M., Sachs M.C., Zhou Y., Monsell S.E., Saver J.L., Vinters H.V. (2014). Clinical predictors of severe cerebral amyloid angiopathy and influence of APOE genotype in persons with pathologically verified Alzheimer disease. JAMA Neurol..

[76] Sanchez-Caro J.M., de Lorenzo Martínez de Ubago I., de Celis Ruiz E., Arribas A.B., Calviere L., Raposo N., Blancart R.G., Fuentes B., Diez-Tejedor E., Rodriguez-Pardo J. (2022). Transient Focal Neurological Events in Cerebral Amyloid Angiopathy and the Long-term Risk of Intracerebral Hemorrhage and Death: A Systematic Review and Meta-analysis. JAMA Neurol..

[77] Catak C., Zedde M., Malik R., Janowitz D., Soric V., Seegerer A., Krebs A., Düring M., Opherk C., Linn J. (2019). Decreased CSF Levels of ß-Amyloid in Patients With Cortical Superficial Siderosis. Front. Neurol..

[78] Ly J.V., Singhal S., Rowe C.C., Kempster P., Bower S., Phan T.G. (2015). Convexity Subarachnoid Hemorrhage with PiB Positive Pet Scans: Clinical Features and Prognosis. J. Neuroimaging.

[79] Tsai H.H., Pasi M., Liu C.J., Tsai Y.C., Yen R.F., Chen Y.F., Jeng J.S., Tsai L.K., Charidimou A., Baron J.C. (2025). Differentiating Cerebral Amyloid Angiopathy From Alzheimer’s Disease Using Dual Amyloid and Tau Positron Emission Tomography. J. Stroke.

[80] Zedde M., Napoli M., Moratti C., Pezzella F.R., Seiffge D.J., Tsivgoulis G., Caputi L., Salvarani C., Toni D., Valzania F. (2024). The Hemorrhagic Side of Primary Angiitis of the Central Nervous System (PACNS). Biomedicines.

[81] Pascarella R., Antonenko K., Boulouis G., De Boysson H., Giannini C., Heldner M.R., Kargiotis O., Nguyen T.N., Rice C.M., Salvarani C. (2023). European Stroke Organisation (ESO) guidelines on Primary Angiitis of the Central Nervous System (PACNS). Eur. Stroke J..

[82] Scolding N.J., Joseph F., Kirby P.A., Mazanti I., Gray F., Mikol J., Ellison D., Hilton D.A., Williams T.L., MacKenzie J.M. (2005). Abeta-related angiitis: Primary angiitis of the central nervous system associated with cerebral amyloid angiopathy. Brain.

[83] Grangeon L., Boulouis G., Capron J., Bala F., Renard D., Raposo N., Ozkul-Wermester O., Triquenot-Bagan A., Ayrignac X., Wallon D. (2024). Cerebral Amyloid Angiopathy-Related Inflammation and Biopsy-Positive Primary Angiitis of the CNS: A Comparative Study. Neurology.

[84] Panteleienko L., Banerjee G., Mallon D.H., Harvey V., Oliver R., Hotton G., Knight W., Datta S., Zandi M.S., Jäger H.R. (2024). Sulcal Hyperintensity as an Early Imaging Finding in Cerebral Amyloid Angiopathy-Related Inflammation. Neurology.

[85] Cai L., Tozer D.J., Markus H.S. (2025). Cerebral Microbleeds and Their Association With Inflammation and Blood-Brain Barrier Leakage in Small Vessel Disease. Stroke.

[86] Ahn S.J., Anrather J., Nishimura N., Schaffer C.B. (2018). Diverse Inflammatory Response After Cerebral Microbleeds Includes Coordinated Microglial Migration and Proliferation. Stroke.

[87] Rosidi N.L., Zhou J., Pattanaik S., Wang P., Jin W., Brophy M., Olbricht W.L., Nishimura N., Schaffer C.B. (2011). Cortical microhemorrhages cause local inflammation but do not trigger widespread dendrite degeneration. PLoS ONE.

[88] Sumbria R.K., Grigoryan M.M., Vasilevko V., Krasieva T.B., Scadeng M., Dvornikova A.K., Paganini-Hill A., Kim R., Cribbs D.H., Fisher M.J. (2016). A murine model of inflammation-induced cerebral microbleeds. J. Neuroinflammation.

[89] Koemans E.A., Chhatwal J.P., van Veluw S.J., van Etten E.S., van Osch M.J.P., van Walderveen M.A.A., Sohrabi H.R., Kozberg M.G., Shirzadi Z., Terwindt G.M. (2023). Progression of cerebral amyloid angiopathy: A pathophysiological framework. Lancet Neurol..

[90] Kozberg M.G., Yi I., Freeze W.M., Auger C.A., Scherlek A.A., Greenberg S.M., van Veluw S.J. (2022). Blood-brain barrier leakage and perivascular inflammation in cerebral amyloid angiopathy. Brain Commun..

[91] Greenberg S.M., Bax F., van Veluw S.J. (2025). Amyloid-related imaging abnormalities: Manifestations, metrics and mechanisms. Nat. Rev. Neurol..

[92] Makkinejad N., Zanon Zotin M.C., van den Brink H., Auger C.A., Vom Eigen K.A., Iglesias J.E., Greenberg S.M., Perosa V., van Veluw S.J. (2024). Neuropathological Correlates of White Matter Hyperintensities in Cerebral Amyloid Angiopathy. J. Am. Heart Assoc..

[93] van Veluw S.J., Benveniste H., Bakker E.N.T.P., Carare R.O., Greenberg S.M., Iliff J.J., Lorthois S., Van Nostrand W.E., Petzold G.C., Shih A.Y. (2024). Is CAA a perivascular brain clearance disease? A discussion of the evidence to date and outlook for future studies. Cell. Mol. Life Sci..

[94] Auger C.A., Perosa V., Greenberg S.M., van Veluw S.J., Kozberg M.G. (2023). Cortical superficial siderosis is associated with reactive astrogliosis in cerebral amyloid angiopathy. J. Neuroinflammation.

[95] Ohashi S.N., DeLong J.H., Kozberg M.G., Mazur-Hart D.J., van Veluw S.J., Alkayed N.J., Sansing L.H. (2023). Role of Inflammatory Processes in Hemorrhagic Stroke. Stroke.

[96] Charidimou A., Perosa V., Frosch M.P., Scherlek A.A., Greenberg S.M., van Veluw S.J. (2020). Neuropathological correlates of cortical superficial siderosis in cerebral amyloid angiopathy. Brain.

[97] Koemans E.A., van Walderveen M.A.A., Voigt S., Rasing I., van Harten T.W., JA van Os H., van der Weerd N., Terwindt G.M., van Osch M.J.P., van Veluw S.J. (2023). Subarachnoid CSF hyperintensities at 7 tesla FLAIR MRI: A novel marker in cerebral amyloid angiopathy. Neuroimage Clin..

